# Novel compound heterozygous *ALPK3* mutations (c.4234C>T and c.3491G>A), causing hypertrophic cardiomyopathy treated with the liwen procedure: case report

**DOI:** 10.3389/fcvm.2025.1671882

**Published:** 2025-12-08

**Authors:** Wen-Jing Liu, Yang Hua, Feng-Hui Jiao, Hai-Ying Hu, Wen-Juan Xu, Ya-Nan Wang, Li-Ping Duan, Xiu-Feng Zhao, Ren-Jie Zhang, Chao Chang

**Affiliations:** 1 Department of Cardiology I, The First Hospital of Handan City, Handan, China; 2 Computed Tomography Suite, Wei County Hospital of Traditional Chinese Medicine, Wei County, China

**Keywords:** hypertrophic cardiomyopathy, occult biventricular obstructive hypertrophic cardiomyopathy, *ALPK3* gene, domain architecture and mutation distribution of *ALPK3*, liwen procedure

## Abstract

**Background:**

Hypertrophic cardiomyopathy (HCM) is an autosomal dominant cardiovascular disease characterised by myocardial hypertrophy with a prevalence of approximately 0.2%–0.5%. Recently, in addition to mutations in genes encoding sarcomeric proteins, which have traditionally been implicated in the development of HCM, mutations in genes encoding non-sarcomeric proteins have also been found to be associated with the development of HCM.This report details the first documented case in China of severe HCM caused by compound heterozygous mutations in the non-sarcomeric proteins *Alpha-kinase 3* (*ALPK3*) gene.

**Case presentation:**

This article reports the case of an 18-year-old female patient with HCM, who presented to hospital with sudden transient loss of consciousness while hiking,and the diagnosis was confirmed by echocardiography and genetic testing. Whole exome sequencing revealed a novel compound heterozygous variant in the *ALPK3* gene (c.4234C > T nonsense mutation and c.3491G > A missense mutation) in the proband, which was reported for the first time in China. The patient presented with severe myocardial hypertrophy, biventricular involvement, occult biventricular obstruction, simian crease, history of syncope and high risk of sudden death. After ineffective conservative pharmacological treatment, the patient underwent the first international percutaneous intramyocardial septal radiofrequency ablation (PIMSRA, Liwen procedure), which resulted in complete remission of clinical symptoms 6 months after the procedure. It strongly supports the consideration of the Liwen procedure as an effective therapeutic strategy for similar patients harboring pathogenic *ALPK3* variants.

**Conclusions:**

This case suggests that compound heterozygosity for nonsense mutations combined with missense mutations in the *ALPK3* gene can lead to early-onset severe HCM, enriches the mutation spectrum of the *ALPK3* gene, reveals high frequency mutation sites in exons 4 and 10 specific to East Asian populations, suggesting potential racial genetic heterogeneity, and that the Liwen procedure is a safe and effective treatment for HCM.

## 1 Introduction

Hypertrophic cardiomyopathy(HCM) is the most common autosomal dominant cardiovascular disease, mainly due to a pathogenic variant of the gene encoding sarcomeric proteins, or a cardiomyopathy characterised by myocardial hypertrophy of unknown etiology, the need to exclude other cardiovascular diseases or systemic, metabolic diseases caused by ventricular wall thickening, echocardiography or magnetic resonance examination of the left ventricle at end-diastole in any part of the ventricular wall thickness ≥15 mm. The diagnosis can be confirmed by a positive test for the causative gene or by examining members of genetically affected families with LV wall thickness ≥13 mm (1), and the prevalence of the disease is approximately 1/500–1/200 (2, 3), with causative or potentially causative genetic variants present in approximately 60% of cases and no clear causative gene identified in approximately 40% of cases of HCM (4).

It is now increasingly recognised that HCM has a complex genetic aetiology, in HCM patients, over 90% of the harmful genetic variants are due to the eight core genes that encode myelin. However, variants in genes encoding non-sarcomeric proteins with diverse functions, such as *ACTN2, ALPK3, CSRP3, FHOD3*, and *FLNC*, have been identified as causative in a minority of patients (5).

The gene has been mapped to chromosome 15q25.2 and contains 14 exons, *ALPK3* is an atypical protein kinase family that recognises phosphorylation sites in the alpha-helix. It can broadly regulate cell migration, adhesion and proliferation, vesicular transport and protein translation (6). *ALPK3* gene mutations tend to be autosomal recessive (7), Lopes et al. first reported the existence of dominant inheritance of the *ALPK3* gene in 2021, and the autosomal dominant mode of inheritance is often dominated by truncating variants (8), patients who carry pathogenic variants of the *ALPK3* gene may present with varying degrees of cardiac hypertrophy or dilated cardiomyopathy, heart failure and, in some cases, a combination of non-cardiac symptoms such as short stature, peculiar facial features, cleft palate, short neck, scoliosis, knee and shoulder contractures (9, 10), heterozygous mutations are characterised by significant phenotypic heterogeneity, they are particularly rare in East Asian populations (11).

Recently, our centre found the first case of *ALPK3* compound heterozygous mutation (c.4234C > T nonsense mutation and c.3491G > A missense mutation) in HCM in China, and the patient underwent percutaneous intramyocardial septal radiofrequency ablation (PIMSRA, Liwen procedure). The patient recovered well after the procedure, and the 6-month follow-up showed that the septal thickness decreased from 29 mm to 15 mm, and the left ventricular end-diastolic diameter (LVDd) increased to 45 mm, and the clinical symptoms were completely relieved. This case broadens the mutational spectrum of the *ALPK3* gene and provides compelling evidence supporting the efficacy of the Liwen procedure (a pioneering minimally invasive technique developed in China) for treating drug-refractory HCM. Notably, this intervention significantly improved the clinical outcome in this patient with severe ALPK3-related HCM. Additionally, analysis of *ALPK3* pathogenic variant distribution identified exons 4 and 10 as frequent mutation sites in East Asian populations, suggesting a possible population-specific pattern in *ALPK3*-related pathogenicity.

## 2 Case presentation

### 2.1 History of illness and physical examination

**Figure 1:**
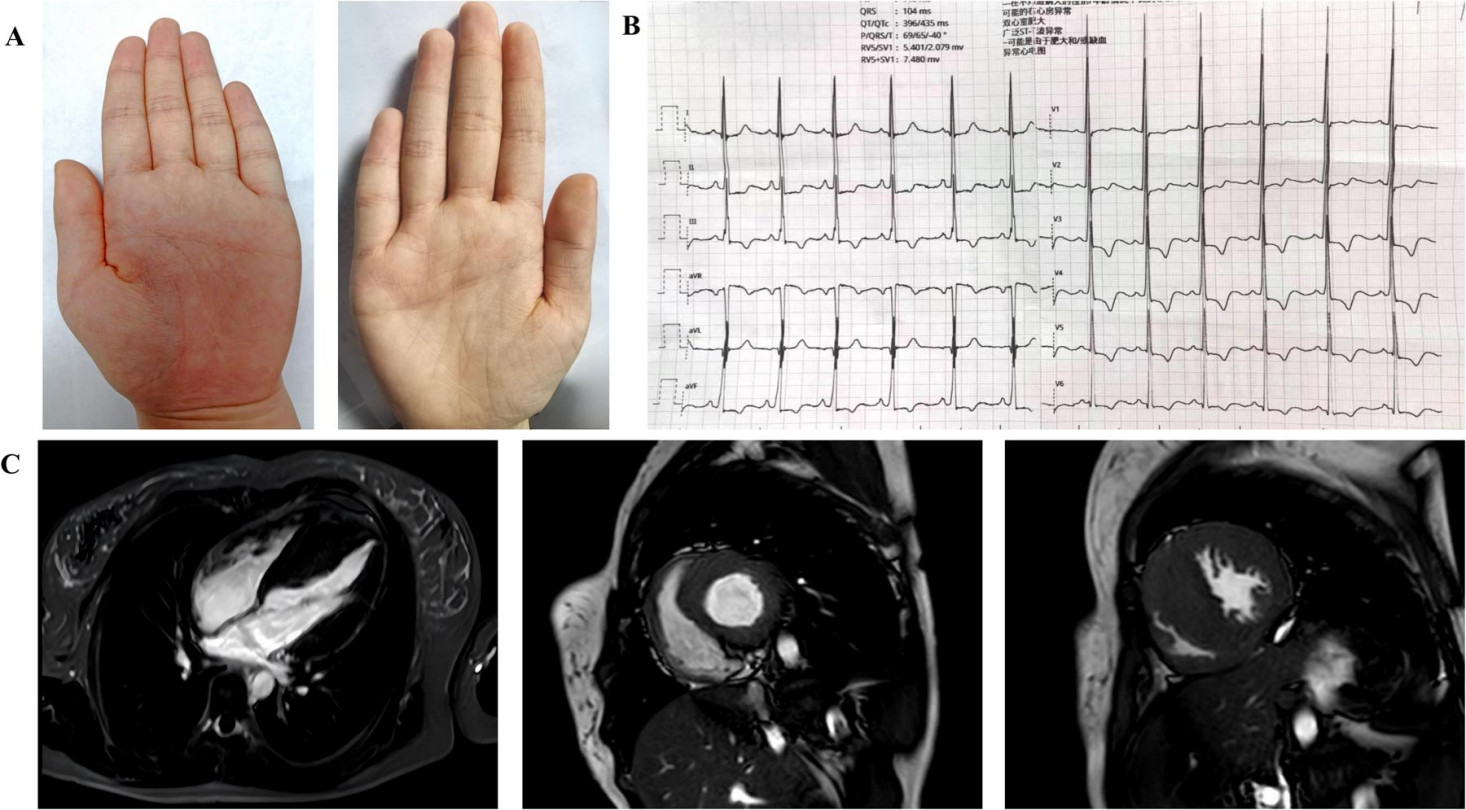
**(A)** Extracardiac manifestations in patients with compound heterozygous mutations in the *ALPK3* gene: palms with simian creases in both hands. **(B)** Electrocardiogram. The electrocardiogram showed left ventricular hypervoltage and myocardial ischemia. **(C)** Preoperative cardiac magnetic resonance: Four-chamber view of the heart; Short-axis views of the left ventricle (end diastole). Diffuse hypertrophy of the left ventricle and thickening of the RVFW were observed. End-diastolic measurements revealed an IVS thickness of 29 mm and an RVFW thickness of 8 mm. Late gadolinium enhancement was absent in the septum and the mid-to-distal segments of the left ventricle.

The patient is a female, 18 years old, with symptoms of chest tightness and shortness of breath during activities in June 2023, and sudden loss of consciousness while hiking in December 2023 (lasting 1 min), and in June 2024, she was seen at the Department of Cardiology, First Hospital of Handan City. Physical examination: height 156 cm, weight 54.5 kg, normal development, normal intelligence, simian crease (Figure 1A), no enlargement of cardiac borders, no pathological murmur in the precordial region, and no other extracardiac signs:such as short neck, scoliosis, knee and shoulder contractures. There was no family history of sudden death or cardiomyopathy.

### 2.2 Laboratory and imaging evaluation

Laboratory tests: N-terminal proB-type natriuretic peptide (NT-proBNP) level 4,015 pg/mL (reference value <125 pg/mL); Cardiac troponin T level 12 pg/mL (reference value <14 pg/mL); and no abnormalities were seen in blood routine; urine routine;liver function; kidney function; coagulation function; thyroid function;and cardiac enzymes.

Electrocardiogram: A 12-lead electrocardiogram suggests left ventricular hypervoltage and myocardial ischemia (Figure 1B). A 24-hour ambulatory electrocardiogram was performed, but no malignant arrhythmias were detected.

Transthoracic echocardiography demonstrated increased myocardial thickness with an interventricular septum (IVS) of 29 mm and a right ventricular free wall (RVFW) measuring 8 mm. The LVDd was 37 mm with a left ventricular end-diastolic volume (LVEDV) of 56.9 mL. At rest, the left ventricular outflow tract pressure gradient (LVOT-PG) is 5.6 mmHg (Vmax 119 cm/s) and in the left ventricular intracavitary compartment pressure gradient (LVIC-PG) is 4.3 mmHg (Vmax 104 cm/s), the right ventricular outflow tract pressure gradient (RVOT-PG) exhibited elevated resting gradients at 18 mmHg (Vmax 214 cm/s). Following provocation with exercise echocardiography, significant dynamic increases were observed: LVOT-PG escalated to 20 mmHg (Vmax 224 cm/s), LVIC-PG rose to 31 mmHg (Vmax 280 cm/s), and RVOT-PG reached 28 mmHg (Vmax 261 cm/s). These findings collectively support the diagnosis of occult biventricular obstructive HCM.

Cardiac magnetic resonance showed asymmetric hypertrophy of the IVS(thickness of 29 mm) and thickening of the RVFW (8 mm), and late gadolinium enhancement was negative(Figure 1C).

Coronary CTA: There is no stenosis or malformation of the coronary arteries.

### 2.3 Whole exome sequencing

A peripheral blood sample was collected from the patient in an EDTA anticoagulant tube and kept at 4°C for under 6 h. DNA was extracted using the Blood Genome Column Medium Extraction Kit (Tiangen Biotech, Beijing, China) as per the instructions. Using the SureSelectXT Clinical Research Exome V4, the protein-coding exome was enriched. Whole exome sequencing (WES) was conducted on the Illumina Hiseq X10 platform (Illumina, San Diego, CA, USA), with initial quality control carried out using FastP to process raw data and eliminate low-quality reads.Variants were annotated based on minor allele frequencies from databases and the American College of Medical Genetics’ practical guidelines on pathogenicity. The pathogenicity of variants was predicted using MutationTaster software and CADD scaled c-scores, with the GRCh37 reference genome serving for alignment. We examined databases such as gnomAD, ExAC, and 1,000 G to determine the prevalence of variants.

**Figure 2:**
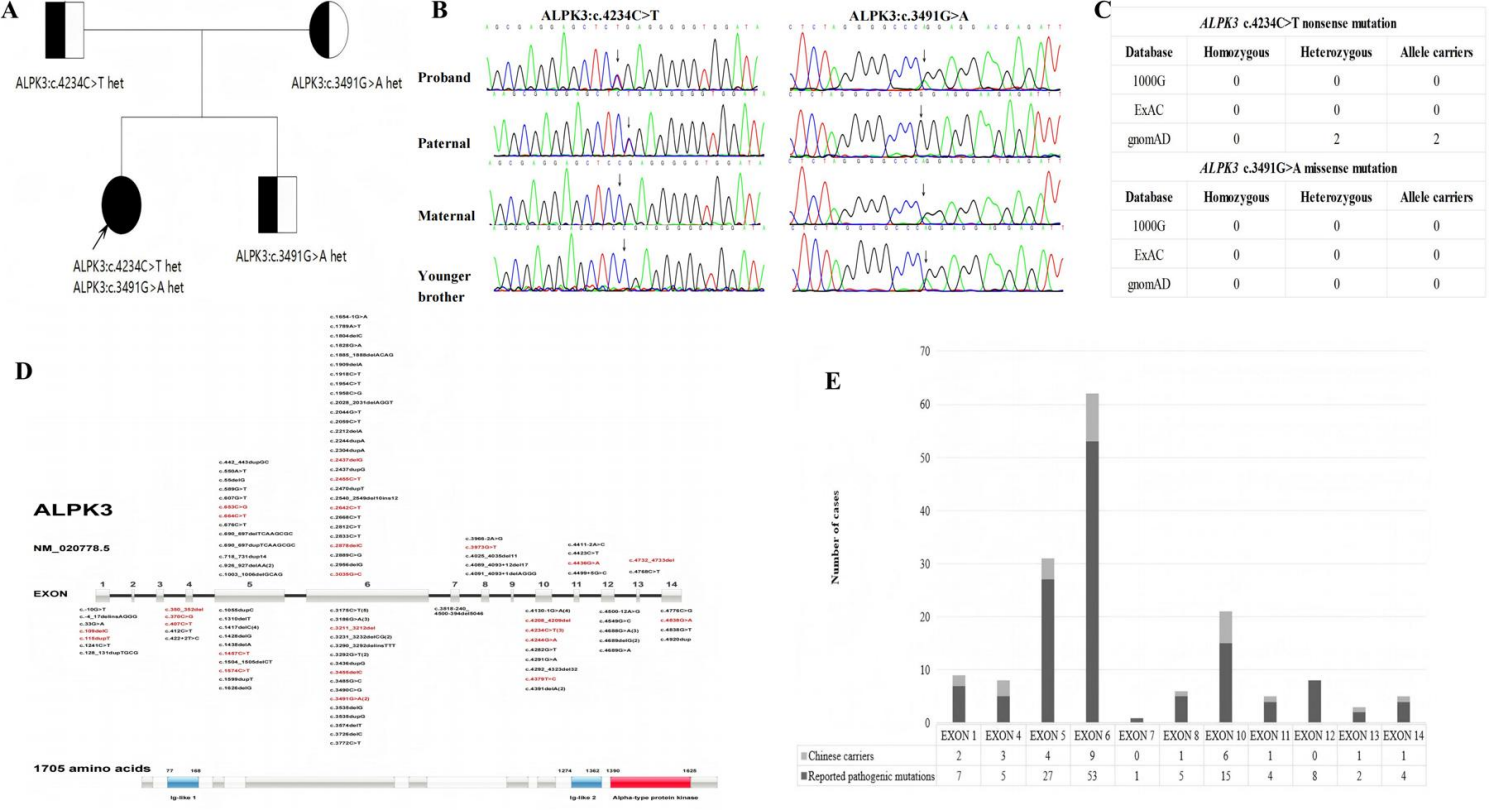
**(A)** family tree of *ALPK3* gene mutation. □ Male, ○ Female, ● Patient, 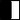 Carrier, 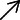 Proband. **(B)** Sanger sequencing of the proband's first-degree relatives.**(C)** Variant frequency analysis of the *ALPK3* mutations (c.4234C > T nonsense and c.3491G > A missense) across three major population genomic databases (gnomAD, ExAC, 1,000 Genomes Project) demonstrated.**(D)** The domain architecture and mutation distribution of *ALPK3:*Distribution of pathogenic variants in *ALPK3* across populations in China and abroad. Red indicates carriers in the Chinese population, while black indicates carriers in non-Chinese populations.**(E)** Pathogenic variants in the *ALPK3* gene exhibit specific distribution characteristics within human populations. Pathogenic mutations in this gene primarily occur in exons 5, 6, and 10. Variations in these three exons account for 72.52% of all reported cases. Variations in exon 4 (60%) and exon 10 (40%) are more prevalent in the Chinese population than the global average, suggesting potential racial specificity.

Genetic analysis revealed that the proband carried a compound heterozygous mutation in the *ALPK3* gene. His immediate family members underwent Sanger sequencing for the paternally derived nonsense mutation *ALPK3*: c.4234C > T (p.Arg1412Ter) and the maternal missense mutation c.3491G > A (p.Arg1164Gln). According to the American College of Medical Genetics (ACMG) guidelines for mutation classification, the mutations c.4234C > T (p.Arg1412Ter) were categorized as pathogenic variants (PVS1 + PM2 + PM3) and c.3491G > A (p.Arg1164Gln) was classified as a variant of undetermined significance (PM2 + PP3). However, the c.3491G > A variant has since been reported in a Turkish HCM patient, where it was identified in a compound heterozygous state with the p.Ser653Ter variant (12). This evidence bolsters the plausibility that the c.3491G > A variant, likely resulting in a loss of function, contributes to the disease phenotype in a compound heterozygous state with the p.Arg1412Ter variant. The brother carries the ALPK3 c.3491G > A (p.Arg1164Gln), the parents and brother are single heterozygous carriers with a normal phenotype, the proband was compound heterozygous for both variants and manifested HCM, the grandparents refused the genetic test (Figures 2A,B).

Variant frequency analysis of the *ALPK3* mutations (c.4234C > T nonsense and c.3491G > A missense) across three major population genomic databases (gnomAD, ExAC, 1,000 Genomes Project) demonstrated: The c.4234C > T nonsense variant was observed in 2 heterozygous carriers exclusively in gnomAD, with no occurrences in other databases. No homozygous or heterozygous carriers of the c.3491G > A missense variant were detected in any database. These robust findings provide compelling evidence that the compound heterozygous genotype (c.4234C > T and c.3491G > A) represents the first reported pathogenic variant combination associated with early-onset severe HCM in China. However, it requires further follow-up observation to determine whether the *ALPK3* gene c.4234C > T nonsense mutation or the *ALPK3* gene c.3491G > A missense mutation alone leads to HCM (Figure 2C).

### 2.4 The domain architecture and mutation distribution of *ALPK3*

Distribution of *ALPK3* disease-causing variants: A database search using the terms “*ALPK3* gene” (database established until April 2025) revealed that 131 *ALPK3* mutation sites have been reported worldwide, of these, 72.52% are concentrated in exons 5, 6, and 10. The distribution of mutations in the Chinese population was consistent with the global pattern; however, the detection rates for exons 4 and 10 were significantly higher (60% and 40%, respectively), suggesting potential race specificity. However, due to the small number of cases in China (*n* = 28), regional differences in detection rates require verification by increasing the sample size (Figures 2D,E).

### 2.5 Treatment and follow-up

In this case, the patient presented with biventricular hypertrophy, early-onset heart failure, chest tightness during daily activities, and cardiac function class III (New York Heart Association, NYHA) classification, and the patient also had a history of syncope. During exercise echocardiography, the patient achieved the target heart rate (171 bpm) using a standardized protocol (3 stages with a 25W/3 min increment). The peak gradient was observed at 1 min of recovery, hemodynamic measurements demonstrated a LVOT-PGmax of 20 mmHg, an LVIC-PGmax of 31 mmHg, and a RVOT-PGmax of 28 mmHg, based on these hemodynamic findings during physiological provocation, the patient was diagnosed with occult left ventricular intracavitary obstruction accompanied by concurrent RVOT obstruction.

Given the proband's status as a young female with high functional demand, the patient and family jointly opted for the Liwen procedure to minimize disruption to daily activities and academic pursuits. The Liwen procedure™ was performed under real-time echocardiographic guidance without cardioplegic arrest, a radiofrequency ablation needle was percutaneously advanced through the intercostal space and cardiac apex to target hypertrophic septal myocardium, high-frequency alternating current from the needle tip induced localized hyperthermia, myocardial dehydration, and irreversible coagulative necrosis, concurrent ablation of septal branches interrupted vascular supply to hypertrophic tissue, reducing septal thickness and relieving obstruction. On July 10, 2024, the patient underwent this procedure under general anesthesia in the left lateral decubitus position (right shoulder elevated 30°), with continuous electrocardiographic and echocardiographic monitoring, following ultrasound-guided localization of the puncture site, the Cool-tip™ ACT-2020 radiofrequency needle was advanced along the long axis of the interventricular septum to hypertrophic regions, sequential ablation was performed at: the basal septum (Zones I, II, III), mid-ventricular septum (Zones I, II, III) and the apical region, using maximum power outputs of 70W, 80W, and 60W for each respective zone(mean duration: 10 min/zone), a total of 7 ablations were performed (13, 14). The procedure was completed without any intraoperative or postoperative complications.

**Figure 3:**
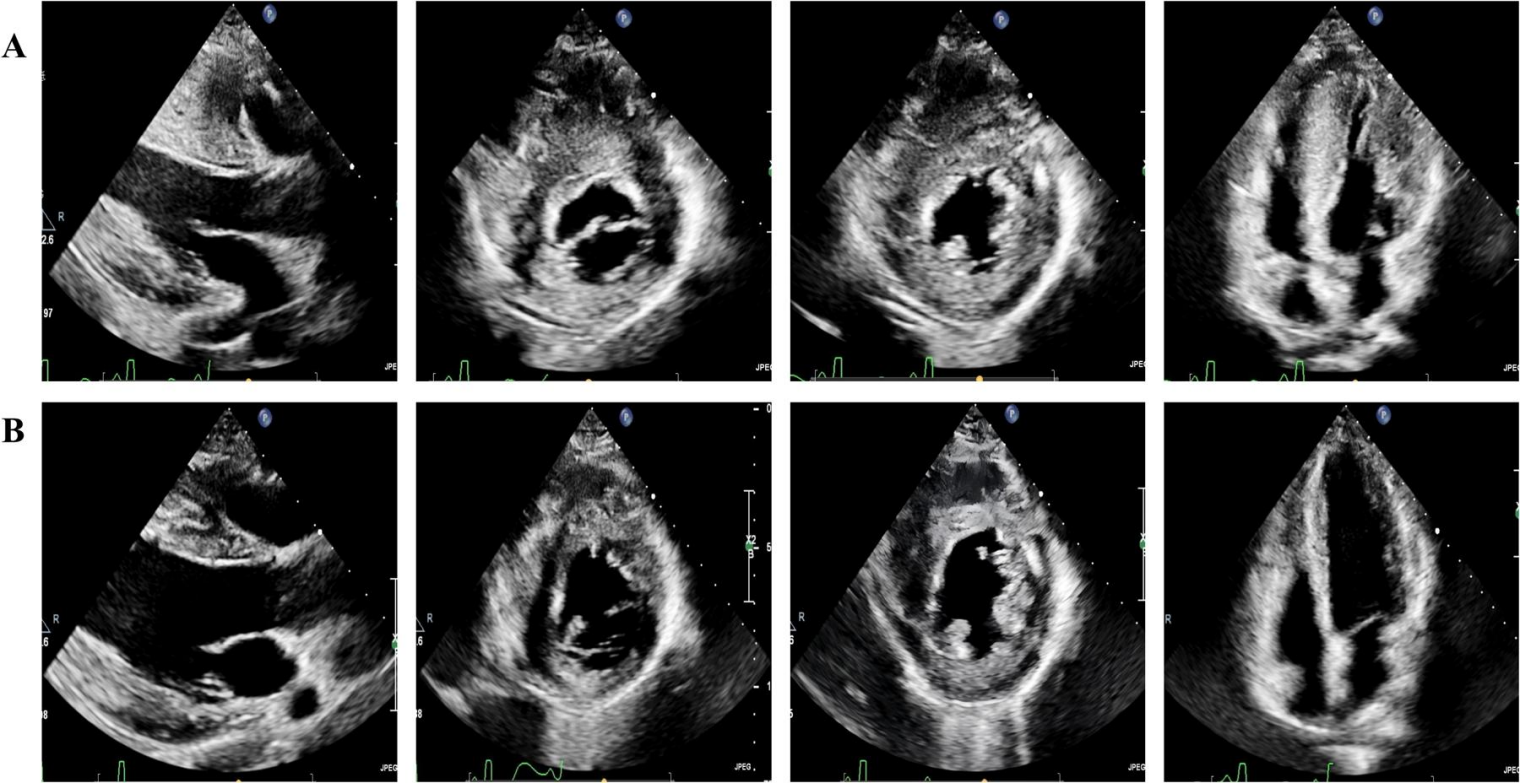
Transthoracic echocardiography. **(A)** Preoperative transthoracic echocardiography demonstrates severe left ventricular hypertrophy, right ventricular free wall hypertrophy, and a small LVEDV in parasternal long axis of left ventricule, parasternal short axis, and apical four-chamber view. **(B)** A 6-month postoperative transthoracic echocardiography, compared with **(A)**, reveals significant thinning of both the left ventricular and right ventricular free wall myocardium, alongside a marked increase in LVEDV.

Transthoracic echocardiography was performed during the 6-month follow-up period after the procedure,septal thickness decreased from 29 mm to 15 mm, the LVDd increased from 37 mm to 45 mm and an increased from 56.9 mL to 101 mL was seen in the LVEDV (Figures 3A,B), left ventricular mass and hemodynamic measurements were significantly reduced, NT-proBNP was 2,233 pg/mL, the patient exhibited excellent clinical recovery with complete resolution of exertional symptoms, including chest tightness and dyspnea, there was also an absence of syncopal episodes, detailed data are shown in Table 1.

**Table 1:** Comparison of patient parameters.

Variable	Pre-operative	6-month post operative
IVS (thickness)(mm)	29	15
RVFW (mm)	8	7
LVDd (mm)	37	45
LVEDV (mL)	56.9	101
LVM (g)	182	120
LVMI (g/㎡)	130	79.2
LVOT (rest) (mmHg)	5.6	8.2
LVOT (provocation) (mmHg)	20	4.9
LVIC (rest) (mmHg)	4.8	7
LVIC (provocation) (mmHg)	31	1.8
RVOT (rest) (mmHg)	18	4.4
RVOT (provocation) (mmHg)	28	4.7
NT-proBNP (pg/mL)	4,457	2,233

## 3 Discussion

Sarcomeric gene mutations predominate in patients with HCM, with pathogenic variants in *MYBPC3* (60.5%) and *MYH7* (14.1%) collectively accounting for 74.6% of mutation-positive cases (15), recent advances have identified *ALPK3*, a non-sarcomeric protein-coding gene, as a novel pathogenic contributor to HCM. However, the precise molecular mechanisms remain incompletely characterized. Current evidence suggests that *ALPK3* variants may induce cardiomyopathic changes through four distinct pathophysiological mechanisms: (1) disruption of myosin localization, leading to structural and functional impairment of cardiomyocytes that culminates in cellular hypertrophy (16); (2) dysregulation of HEY2 transcription factor phosphorylation and consequent abnormalities in cardiomyocyte differentiation (17); (3) induction of calcium homeostasis dysregulation, thereby elevating arrhythmogenic risk (5); (4) manifestation of sarcomeric disarray and aberrant intercalated disc morphology, resulting in contractile dysfunction (18).

Multicenter international have demonstrated that *ALPK3* truncating variants account for 1%–2% of HCM cases. These variants are characterized by later disease onset and predominantly non-obstructive phenotypes at rest, these variants typically present with apical or concentric hypertrophy and are often accompanied by extracardiac manifestations. Notably, heterozygotes exhibit significant clinical heterogeneity (8), and a Portuguese cohort study confirmed that *ALPK3* mutations constitute 2.7% of HCM etiologies (19), interestingly, East Asian populations have distinct genetic profiles in HCM pathogenesis (11). Both the first Chinese case reported by our center and the East Asian study found that compound heterozygous mutations (e.g., in this case, c.4234C > T nonsense mutation and c.3491G > A missense mutation) could lead to a more severe clinical phenotype manifested by early-onset biventricular hypertrophy, multilevel outflow tract obstruction, and simian crease.

The *ALPK3* gene encodes nuclear alpha-protein kinase 3, which is critical for cardiomyocyte development and structural homeostasis, reported pathogenic variants primarily consist of nonsense and missense mutations and splice-site variations, with biallelic or compound heterozygous configurations predominantly observed (12, 20). Studies indicate that nonsense mutations induce premature protein truncation, potentially exerting pathogenic effects through haploinsufficiency or dominant-negative mechanisms; Meanwhile, missense mutations are postulated to compromise kinase domain functionality by disrupting critical structural motifs (12); additionally, splice-site variants may impair protein expression through aberrant mRNA splicing processes that alter transcriptional integrity (21). Wei-Feng et al. demonstrated that *ALPK3* double allele deletion can trigger lethal cardiomyopathy in an animal model study, approximately 75% of germline knockout mice die within one month of birth, and survivors exhibit a transformation from dilated cardiomyopathy to HCM, which is highly consistent with the clinical manifestations of compound heterozygous mutations in humans. Studies have demonstrated that *ALPK3* acts as a scaffolding protein that regulates thick filament protein turnover (22). In animal models, *ALPK3* knockout mice exhibit a mixed phenotype of hypertrophic and dilated cardiomyopathy, suggesting that *ALPK3* plays a critical role in the development and function of cardiomyocytes (23), these findings imply that *ALPK3* gene mutations may cause structural and functional abnormalities in cardiomyocytes and subsequently lead to cardiomyopathy.

This HCM case presents compound heterozygosity for an *ALPK3* nonsense mutation (c.4234C > T) coupled with a missense mutation (c.3491G > A), compared to *ALPK3* heterozygotes, compound heterozygous carriers exhibit an aggressive clinical profile characterized by the following: (1) early disease onset, (2) biventricular hypertrophy with multilevel obstruction, (3) an elevated risk of sudden cardiac death, and (4) accelerated heart failure progression. Notably, the proband exhibited bilateral simian creases, a distinctive extracardiac feature, genetic segregation analysis revealed unaffected heterozygous parents and younger brother with normal echocardiographic parameters, this inheritance pattern strongly supports the biallelic dosage effect theory, and the observed genotype-phenotype correlation is particularly prominent in East Asian cohorts.

The therapeutic objectives for HCM focus on alleviating symptoms, improving cardiac function, and slowing disease progression, although conventional pharmacotherapy (beta-blockers, calcium channel blockers and disopyramide) provides symptomatic relief, it does not address the underlying pathological substrate of myocardial hypertrophy. Current management algorithms incorporate 3 main approaches: (1) pharmacological optimisation for diastolic dysfunction and arrhythmia control, (2) surgical myectomy (Morrow procedure) for left ventricular cavity augmentation and septal thickness reduction, and (3) interventional approaches to diminish LVOT gradients, decelerate heart failure progression and mitigate the risk of sudden cardiac death (4). The Liwen procedure is a novel, ultrasound-guided, percutaneous, intramyocardial, radiofrequency ablation technique that enables real-time, targeted ablation of hypertrophied myocardium, achieving gradient reduction and functional improvement. A multicentre trial enrolled 244 refractory HCM patients who met stringent criteria: resting/provoked LVOT gradients ≥50 mmHg, persistent symptoms of NYHA Grade II or higher, and failure to respond to pharmacotherapy, among the 200 cases in which the procedure was completed, with a median follow-up of 19 months, the Liwen procedure approach demonstrated comparable safety and efficacy profiles to the Morrow procedure, with a significantly lower incidence of arrhythmias: there was a 2.5% incidence of permanent right bundle branch block and no patients with permanent pacemaker implantation (14). Chinese multicentre data further established the superiority of the Liwen procedure over percutaneous endocardial septal radiofrequency ablation (14, 24), with significantly lower recurrence rates compared to alcohol septal ablation (2% vs. 10%) (7).

The Liwen procedure is an option for patients who have not responded well to medication and who still have a high risk of sudden death, such as those with dyspnea, chest pain, or syncope. Compared with traditional surgery, the Liwen procedure has technical advantages such as not opening the chest, a continuous beating heart, precise localization, unrestricted target blood vessels, less trauma, faster recovery, fewer complications, a significant reduction in IVS thickness, and reduced conduction system damage and other complications. The successful treatment of this case further confirms the Liwen procedure's potential and advantages in treating HCM, this procedure has significant clinical value and broad application prospects.

## 4 Patient perspective

When diagnosed with hypertrophic obstructive cardiomyopathy, I felt crushed by despair. This disease severely limited my daily activities and imposed a constant threat of sudden death. I feared it would disrupt my education and future career. After thorough discussion with my family, I opted for the Liwen procedure. Currently, I can engage in routine activities and attend school normally. This intervention has restored my hope for the future.Written informed consent was obtained for publication.

## 5 Conclusions

This case is the first report of early-onset, severe HCM caused by compound heterozygous mutations (c.4234C > T nonsense mutation and c.3491G > A missense mutation) in the *ALPK3* gene in the Chinese population, this finding enriches the mutation spectrum of the *ALPK3* gene and reveals high-frequency mutation loci in exons 4 and 10 that are unique to the East Asian population, these results suggest potential ethnogenetic heterogeneity, the patient presented with severe hypertrophy of the IVS, biventricular involvement, occult multilevel outflow tract obstruction, and simian creases, confirming the “dose effect” theory of *ALPK3* double-allele mutation.

Following the Liwen procedure, the patient experienced complete resolution of symptoms within 6 months, demonstrating significant clinical improvement in this case of severe, drug-refractory *ALPK3*-related HCM. This outcome supports the consideration of the Liwen procedure as an effective therapeutic strategy for complex obstructive *ALPK3*-related HCM, but the 6-month follow-up is a recognized limitation in evaluating long-term outcomes for this potentially progressive cardiomyopathy. To address this, the patient is enrolled in a dedicated long-term surveillance program. This program will track myocardial thickness, key hemodynamic measures, clinical symptoms, and NT-proBNP levels to elucidate the long-term disease course and treatment response.

## Data Availability

The raw data supporting the conclusions of this article will be made available by the authors, without undue reservation.
