# Supplementary material for: Novel compound heterozygous ALPK3 mutations (c.4234C>T and c.3491G>A), causing hypertrophic cardiomyopathy treated with the liwen procedure: case report
Source: Front Cardiovasc Med. 2025 Dec 8;12:1671882. doi: 10.3389/fcvm.2025.1671882 (PMC12719494; doi:10.3389/fcvm.2025.1671882)
Supplement: Supplementary file 1 [file Datasheet1.pdf]

ALPK3:c.3491G>A

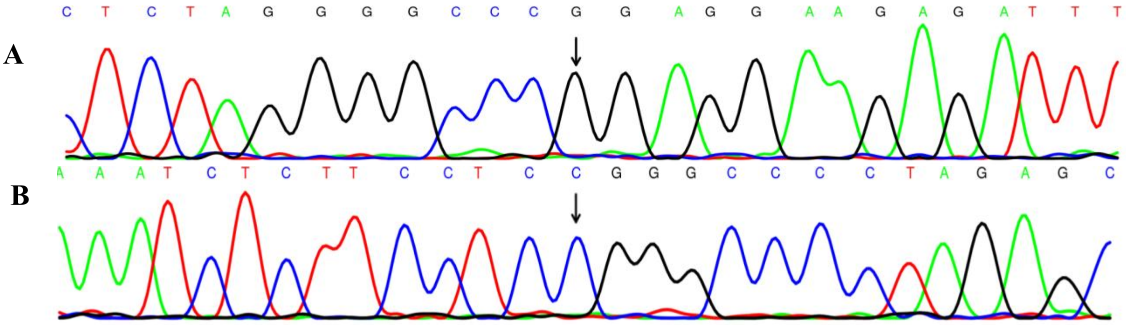

Supplemental Figure 1: Sanger sequencing of the Paternal (A) forward sequencing electropherogram, (B)reverse sequencing electropherogram.

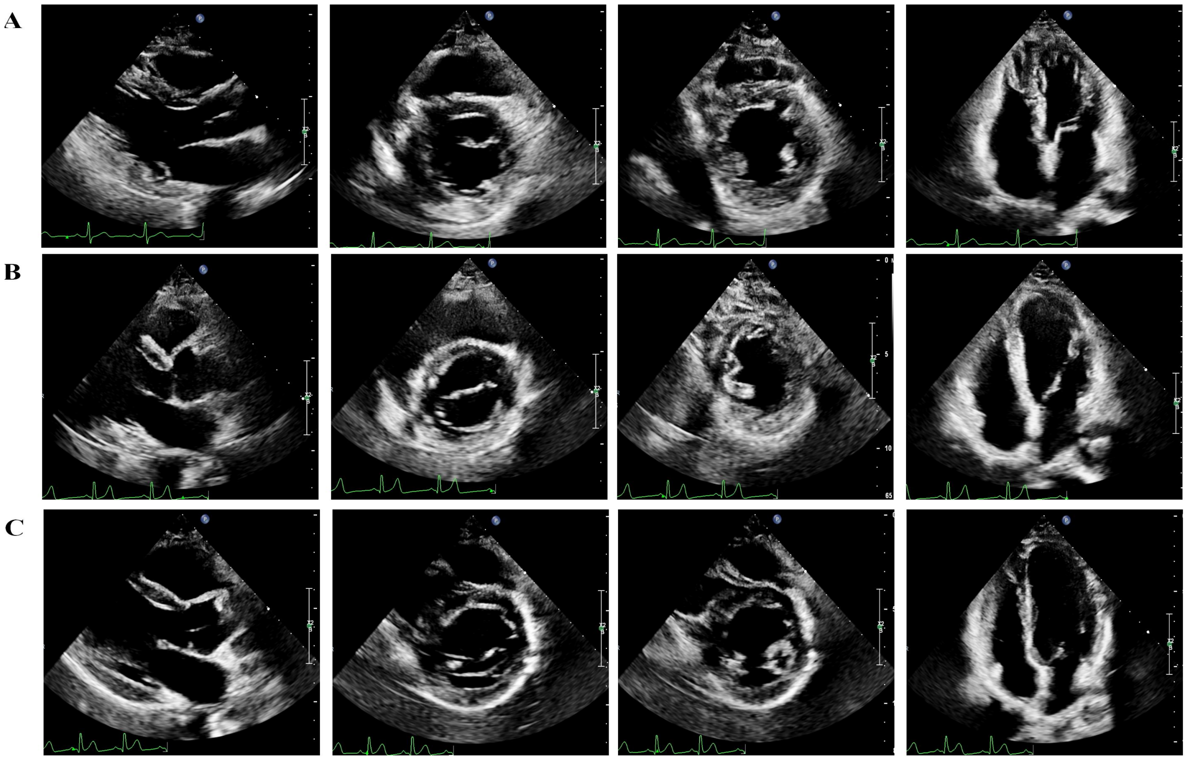

Supplemental Figure 2:Transthoracic echocardiography.Standard views including the left ventricule parasternal long-axis, parasternal short-axis, and apical four-chamber views demonstrate normal myocardial thickness. Images are representative of the (A) paternal, (B) maternal, and (C) younger brother of the proband.

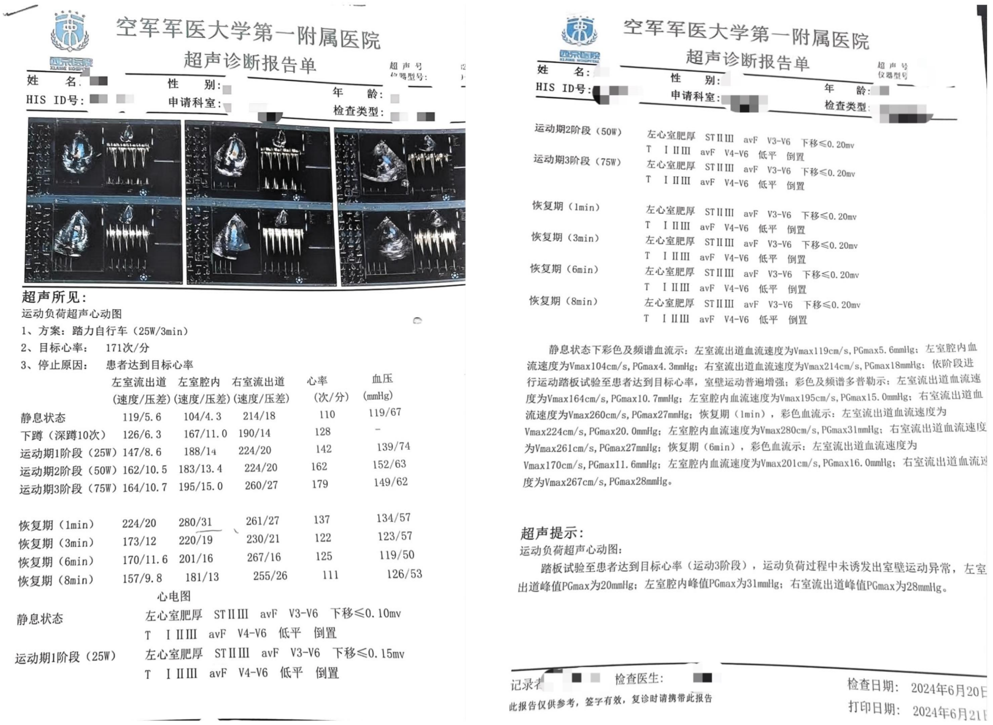

空军军医大学第一附属医院
超声诊断报告单

姓名：[redacted] 性别：[redacted] 超声号 仪器型号
HIS ID号：[redacted] 申请科室：[redacted] 年龄：[redacted] 检查类型：[redacted]

超声所见：
运动负荷超声心动图
1、方案：踏力自行车（25W/3min）
2、目标心率： 171次/分
3、停止原因： 患者达到目标心率

	左室流出道 (速度/压差)	左室腔内 (速度/压差)	右室流出道 (速度/压差)	心率 (次/分)	血压 (mmHg)
静息状态	119/5.6	104/4.3	214/18	110	119/67
下蹲（深蹲10次）	126/6.3	167/11.0	190/14	128	-
运动期1阶段（25W）	147/8.6	188/14	224/20	142	139/74
运动期2阶段（50W）	162/10.5	183/13.4	224/20	162	152/63
运动期3阶段（75W）	164/10.7	195/15.0	260/27	179	149/62
恢复期（1min）	224/20	280/31	261/27	137	134/57
恢复期（3min）	173/12	220/19	230/21	122	123/57
恢复期（6min）	170/11.6	201/16	267/16	125	119/50
恢复期（8min）	157/9.8	181/13	255/26	111	126/53

心电图

静息状态 左心室肥厚 ST II III avF V3-V6 下移≤0.10mv
T I II III avF V4-V6 低平 倒置
运动期1阶段（25W） 左心室肥厚 ST II III avF V3-V6 下移≤0.15mv
T I II III avF V4-V6 低平 倒置

空军军医大学第一附属医院
超声诊断报告单

姓名：[redacted] 性别：[redacted] 超声号 仪器型号
HIS ID号：[redacted] 申请科室：[redacted] 年龄：[redacted] 检查类型：[redacted]

运动期2阶段（50W） 左心室肥厚 ST II III avF V3-V6 下移≤0.20mv
T I II III avF V4-V6 低平 倒置
运动期3阶段（75W） 左心室肥厚 ST II III avF V3-V6 下移≤0.20mv
T I II III avF V4-V6 低平 倒置
恢复期（1min） 左心室肥厚 ST II III avF V3-V6 下移≤0.20mv
T I II III avF V4-V6 低平 倒置
恢复期（3min） 左心室肥厚 ST II III avF V3-V6 下移≤0.20mv
T I II III avF V4-V6 低平 倒置
恢复期（6min） 左心室肥厚 ST II III avF V3-V6 下移≤0.20mv
T I II III avF V4-V6 低平 倒置
恢复期（8min） 左心室肥厚 ST II III avF V3-V6 下移≤0.20mv
T I II III avF V4-V6 低平 倒置

静息状态下彩色及频谱血流示：左室流出道血流速度为Vmax119cm/s，PGmax5.6mmHg；左室腔内血流速度为Vmax104cm/s，PGmax4.3mmHg；右室流出道血流速度为Vmax214cm/s，PGmax18mmHg；依阶段进行运动踏板试验至患者达到目标心率，室壁运动普遍增强；彩色及频谱多普勒示：左室流出道血流速度为Vmax164cm/s，PGmax10.7mmHg；左室腔内血流速度为Vmax195cm/s，PGmax15.0mmHg；右室流出道血流速度为Vmax260cm/s，PGmax27mmHg；恢复期（1min），彩色血流示：左室流出道血流速度为Vmax224cm/s，PGmax20.0mmHg；左室腔内血流速度为Vmax280cm/s，PGmax31mmHg；右室流出道血流速度为Vmax261cm/s，PGmax27mmHg；恢复期（6min），彩色血流示：左室流出道血流速度为Vmax170cm/s，PGmax11.6mmHg；左室腔内血流速度为Vmax201cm/s，PGmax16.0mmHg；右室流出道血流速度为Vmax267cm/s，PGmax28mmHg。

超声提示：
运动负荷超声心动图：
踏板试验至患者达到目标心率（运动3阶段），运动负荷过程中未诱发出室壁运动异常，左室出道峰值PGmax为20mmHg；左室腔内峰值PGmax为31mmHg；右室流出道峰值PGmax为28mmHg。

记录者：[redacted] 检查医生：[redacted] 检查日期：2024年6月20日
此报告仅供参考，签字有效，复诊时请携带此报告 打印日期：2024年6月21日

Supplemental Figure 3: the exercise stress echocardiography:Preoperative exercise stress echocardiography was performed at the First Affiliated Hospital of Air Force Medical University.

Supplemental Table 1：Ultrasonography Data from Family Members

Variable	paternal	maternal	younger brother
IVS(thickness)(mm)	11	10	9
LVOT(rest)(mmHg)	5	7	6
LVMI(g/㎡)	95	72	70
